# ‘You feel like it’s better to just die’: Death-centric stereotypes and stigma contribute to suicide risk for adolescents living with HIV in Malawi

**DOI:** 10.1371/journal.pgph.0005655

**Published:** 2025-12-29

**Authors:** Maria F. Faidas, Bradley N. Gaynes, Steven M. Mphonda, Jeromy Nyirenda, Maureen Matewere, Jack S. Kramer, Laika Maganga, Kazione Kulisewa, Brian W. Pence, Nivedita L. Bhushan, Melissa A. Stockton

**Affiliations:** 1 School of Medicine, University of North Carolina at Chapel Hill, Chapel Hill, North Carolina, United States of America; 2 Department of Psychiatry, University of California San Diego, San Diego, California, United States of America; 3 Departments of Psychiatry and Epidemiology, University of North Carolina at Chapel Hill, Chapel Hill, North Carolina, United States of America; 4 University of North Carolina Project-Malawi, Lilongwe, Malawi; 5 School of Nursing, University of North Carolina at Chapel Hill, Chapel Hill, North Carolina, United States of America; 6 Department of Psychiatry, Kamuzu University of Health Sciences, Blantyre, Malawi; 7 Department of Epidemiology, University of North Carolina at Chapel Hill, Chapel Hill, North Carolina, United States of America; 8 Research Triangle Institute International, Research Triangle Park, Durham, North Carolina, United States of America; 9 Department of Psychiatry, University of Pennsylvania, Philadelphia, Pennsylvania, United States of America; PLOS: Public Library of Science, UNITED STATES OF AMERICA

## Abstract

Suicide is the second leading cause of death for adolescents worldwide. Adolescents living with HIV (ALWH) in Malawi are particularly at risk, due to stigmatization and a high comorbid prevalence of depression. Internalized stigma is an indicator of depression and suicidality, yet we have a limited understanding of the drivers and impacts of internalized stigma. In this analysis, we assessed how death-centric stereotypes and experienced stigma contribute to the development of internalized stigma and elevate suicide risk for ALWH in Malawi. This qualitative study recruited 68 total participants from three government healthcare facilities in Lilongwe, Malawi. We conducted 13 in-depth interviews and 10 focus group discussions with ALWH, caregivers of ALWH, adolescent peers without HIV, schoolteachers, HIV care providers, and mental health providers. We sought to understand how stigma manifests in key parts of an ALWH’s life and contributes to internalized stigma and suicidal ideation. Guided by thematic analysis, we organized the relevant data into four themes: death-centric stereotypes, experienced stigma, internalized stigma, and suicidality. Death-centric stereotypes were identified as HIV’s association with being a death sentence, obstructing one’s future, and weak physical and mental capacity. From these stereotypes originate specific manifestations of experienced stigma, including insults and mockery, reduced social and economic prospects for adulthood, caregiver lack of investment in vital resources, and exclusion from community activities. Stereotypes and experienced stigma contribute to internalized stigma which manifests as low self-esteem, loss of future-oriented mindset, and self-isolation. This stigmatization process, particularly when perpetuated by family, increased suicide risk for ALWH. This study describes how death-centric stereotypes dominate the public discourse around ALWH, influence stigmatizing behaviors towards ALWH that disenfranchise them in their daily lives, and contribute to the development of internalized stigma and suicidality. Suicide screening and prevention programming, with attention to culturally sensitive stigma reduction, is urgently needed for ALWH.

## Introduction

Adolescents living with HIV (ALWH) are at heightened risk of suicide due to the complex psychosocial challenges from living with a highly stigmatized disease and resulting mental health issues [1]. Their unique adolescent developmental stage compounds their risk of suicide, with suicide being the second leading cause of death for adolescents globally [2,3]. Among ALWH worldwide, the prevalence of lifetime suicidal ideation is 25–30% and lifetime suicide attempt is 13% [1,4]. In Sub-Saharan Africa (SSA), the issue of suicidality amongst ALWH is magnified, as it is home to 89% of all adolescents living with HIV [5]. Three-quarters of suicide deaths occur in low- and middle- income countries (LMICs), putting ALWH in SSA at further increased risk [6]. Moreover, ALWH experience elevated depressive symptoms, with reported depression prevalence ranging from 16-41% amongst ALWH in SSA [7]. Considering that 50–90% of suicides are linked to underlying mental disorders, particularly depression [8–10], ALWH and comorbid depression in SSA are in critical need of suicide prevention interventions.

Stigma is a complex social process that devalues and excludes individuals based on characteristics perceived as different or undesirable [11–14]. HIV stigma has long been recognized as a major factor contributing to suicidality and poor mental health [15,16]. In Malawi, where ALWH face similar psychosocial and developmental challenges to ALWH in other SSA countries, an estimated 25% of ALWH exhibit depressive symptoms [17]. Although data is limited in Malawi, roughly 7% of ALWH endorsed suicidal ideation in the previous two weeks on a depression screening [17]. ALWH in Malawi face stigmatization through pervasive death-centric stereotypes and behaviors including gossip, insults and mocking, physical and social distancing, and abuse [18,19]. ‘Death-centric stereotypes’ is a term developed from this study, and refers to pessimistic survival expectations or negative community beliefs regarding an ALWH’s presumed early mortality, and resulting abilities and potential due to their HIV status. Despite widespread antiretroviral therapy (ART) availability contributing to decreased HIV mortality and making HIV a manageable chronic condition, people living with HIV in Malawi remain pessimistic about their life expectancy [20,21]. Additionally, these ALWH face key social health determinants that exacerbate their underlying risk of suicide, such as pervasive poverty, food insecurity, parental loss, and child-headed households [18,22]. This suicide risk is further exacerbated by structural factors, such as scarce mental health and social support services, and lagging suicide prevention efforts. In addition, attempting suicide is still criminalized in Malawi [23], which further perpetuates stigma and discourages help-seeking behavior.

Internalized stigma may play an important role in the context of depression and suicidality [24–26], and reduced HIV care engagement [27–29]. Internalized stigma is the process of adopting negative societal beliefs and discriminatory attitudes and believing them to be true for oneself, which often presents as shame and guilt [14,24,30]. In Malawi, young age, low education levels, and low social support are associated with higher levels of internalized stigma [30,31]. Despite recognition of ALWH’s vulnerability to stigma in Malawi, we have a limited understanding of the process through which ALWH internalize stigma and how this experience may elevate suicide risk. Elucidating key parts of this stigmatization process will help us understand how internalized stigma may contribute to an increased risk of suicide for ALWH in Malawi.

This paper investigates the impact of pervasive death-centric stereotypes and resulting experienced stigma on the development of internalized stigma and suicidality. We draw from a qualitative study that explored experiences of stigma faced by ALWH in Malawi from a wide range of stakeholders. From this study, we identify and discuss key areas for intervention to address internalized stigma and suicide risk, which require action on multiple socioecological levels.

## Methodology

### Ethics statement

The Institutional Review Boards (IRB) of the University of North Carolina at Chapel Hill (IRB #22-0462) and the Malawi National Health Sciences Research Committee (IRB #22201) approved this study. All participants aged 18 years and above provided written informed consent. All participants aged 13–17 years provided written assent with parental written consent. In accordance with NHSRC ethical standards, emancipated minors below age 18, who were legally married or university students, did not require guardian consent. Before study enrollment, RAs engaged participants in a consent comprehension activity to ensure understanding, by asking a series of questions about study design, and risks and benefits of participation. All participants (and guardian if present) received travel reimbursement equivalent to 10 US dollars, the standard compensation from University of North Carolina (UNC) Project-Malawi.

### Study context and design

In this qualitative study, entitled Strategies for Adolescent Reduction of Stigma (STARS), we sought to understand the lived experience of stigma amongst ALWH and depression, and the unique psychosocial challenges produced by this stigma. STARS was conducted under the HIV Engagement and Adolescent Depression Support (HEADS-UP) study [32], a parent study adapting an evidence-based depression counseling intervention (Friendship Bench [33,34]) for ALWH experiencing depressive symptoms in Lilongwe, Malawi. STARS collected interview and focus group discussion data on stigma experiences, to ultimately inform the development of a stigma-reduction intervention as an additional component of the larger counseling intervention being adapted under HEADS-UP. For ease of study coordination and data analysis, STARS study sites and ALWH recruitment were consistent with the procedures of our parent study, HEADS-UP [19].

### Study sites

The study was conducted at Area 18, Area 25, and Kawale Health Centers, three government primary-care health care facilities in peri-urban Lilongwe. The facilities were chosen due to similar staffing levels, services, and ALWH patient volume. Each clinic organizes “Teen Clubs,” a youth-friendly HIV clinic offered every three months on a Saturday. Teen Clubs provide ART refills and viral load testing for ALWH aged 10–19 years, with clubs divided into two age groups (10–14 and 15–19 years). Teen Clubs bolster this medical care by offering physical activities and educational activities on age-appropriate topics, such as ART adherence, HIV transmission, nutrition, and sexual and reproductive health. Some Teen Clubs additionally offer psychosocial support via counseling services.

### Study sample and recruitment

Recruitment and data collection started on October 7, 2023, and ended on January 23, 2024.

#### ALWH participants.

All ALWH present at five Teen Club clinic days in October 2023 were screened for depressive symptoms using the Beck Depression Inventory-II (BDI-II) [35], a 21-item questionnaire used for screening common depressive symptoms such as low mood, anhedonia, sleep and appetite disturbances, and suicidal ideation. ALWH were purposively sampled and eligible to participate if they were: (1) aged 13–19 years; (2) living with HIV; and (3) screened positive for depressive symptoms with a score ≥ 13 on the BDI-II administered by trained clinic staff or research assistants (RAs) [35]. BDI-II scores classify depressive symptoms as minimal (0–13), mild [14–19], moderate [20–28], and severe (29–63) [35]. The BDI-II has been validated for ALWH in Malawi, with a cutoff ≥13 showing 80% sensitivity for detecting depression [17]. Target recruitment for ALWH was 15 participants.

For those meeting eligibility criteria, ALWH were invited for an interview within two weeks, which is the recall period for the BDI-II. Regardless of study enrollment, any ALWH who screened positive for depression or reported suicidal ideation or behaviors was managed using standard-of-care practices at the study facilities, which included same-day referral to a psychosocial counselor or nurse.

Any ALWH who disclosed suicidal ideation on the BDI-II received additional screening via the Tool for Assessment of Suicide Risk for Adolescents (TASR-A) to assess suicide risk [36,37]. The TASR-A assesses for common risk factors of suicide such as substance use, hopelessness, suicide plan, and access to lethal means. Level of immediate suicide risk was determined based on responses, and ALWH deemed to be at high risk of suicide created a safety plan with psychosocial counselors and involved family members when appropriate.

#### Stakeholder participants.

We also purposively sampled participants from five stakeholder populations that are influential in an ALWH’s life and could provide key insights relevant to different life domains such as home, school, community, and healthcare. Stakeholders included (1) caregivers of ALWH, (2) adolescent peers without HIV, (3) schoolteachers of adolescents, (4) HIV care providers, and (5) mental health providers. Target recruitment for each stakeholder population was 12 participants (2 FGDs with 6 participants each).

*Caregivers* were recruited directly during the consent process with ALWH minors or were invited by ALWH aged 18–19 years. Eligibility criteria for caregivers included: (1) age 18 years and above; (2) a trusted individual who was aware of their ALWH’s HIV status; and (3) play a major role in caregiving for their ALWH. *Adolescent peers* were recruited during clinic visits from the Outpatient Department of Area 18, 25, and Kawale Health Centers who were presenting for healthcare needs other than HIV. Eligibility criteria for adolescent peers included: (1) age 13–19 years; (2) no prior HIV diagnosis; and (3) negative depression screen via the BDI-II. *Schoolteachers* were recruited via adolescent peer participants who provided a study flyer to their teacher. Teachers contacted RAs by phone if they were interested in study participation. Eligibility criteria for teachers included: (1) age 18 years and above; (2) teacher for students aged 13–19 years; and (3) employed at a school within the clinic’s catchment areas. *HIV care providers* were eligible to participate if they were (1) age 18 years and above; (2) government employees working at the clinics; and (3) providing HIV care in their role, such as an ART coordinator, nurse, clinician, or clinic leader at the study facilities. *Mental health providers* were eligible to participate if they were (1) age 18 years and above; and (2) providing mental health care at clinic sites, such as psychiatric nurses, psychosocial counselors, or trained Friendship Bench counselors. The research coordinator invited eligible HIV and mental health care providers by phone for study participation.

Prior to conducting the interview or discussion, each participant completed a short demographic form that captured basic information such as age, occupation, highest education level, and food and money insecurity. Food insecurity was assessed by asking, “In the past 12 months, have you been worried about having enough food for you or your family?” Money insecurity was assessed by asking, “In the past 12 months, have you or your household had to borrow money to get by?” Additionally, ALWH were asked questions about their family composition, such as identification of primary caregiver and if both birth parents are living.

### Data collection

We conducted 13 in-depth interviews (IDIs) with ALWH, and 10 focus group discussions (FGDs) total with 5 stakeholder populations. For each stakeholder population, we conducted 2 FGDs with a target of 6 participants per FGD. For the IDIs and FGDs, we developed a semi-structured interview guide to explore experiences of HIV stigma, depression, intersectional stigma (from HIV and depression), and coping strategies (S1 Text and S2 Text). The interview was guided by the What Matters Most (WMM) stigma framework, which theorizes that stigma is felt most acutely when someone is unable to participate in activities that “matter most” to them [38–41]. This theory is used to identify salient cultural values that allow one to achieve “full personhood” in their local world. Based on this framework, interview guides probed about adolescent roles and responsibilities, and how stigma may impede achieving full personhood in key domains of an ALWH’s life. IDIs aimed to elucidate the highly personal experience of stigma through the insider view of an ALWH. FGDs engaged key stakeholders to understand the experience of stigma from an outsider’s perspective, including opinions from possible perpetrators of stigma.

During the data collection period, IDI guides were revised four times during an iterative process based on participant understanding, RA feedback, and question relevance. These revisions did not significantly alter the content of the guides. The IDIs and FGDs lasted between 60–115 minutes and were conducted in Chichewa, the primary national language of Malawi, in a private research room. The interviews were digitally recorded and directly translated into English by RAs who are fluent in both Chichewa and English. All transcripts were reviewed by the interviewer for translation accuracy.

### Qualitative data analysis

Data analysis was conducted via an iterative and collaborative process that involved four steps: 1) reading for content 2) deductive and inductive coding 3) data display to identify emerging themes, and 4) interpretation [42,43]. Transcripts were reviewed as they became available. After closely reading the majority of IDI transcripts, one study team member drafted an initial codebook. Deductive codes were drawn from WMM theory, interview guides, literature review, and prior experience analyzing stigma data from ALWH in Malawi using The Health Stigma and Discrimination Framework [19,44]. Inductive codes were iteratively added based on emerging ideas and relevant topics raised in both IDIs and FGDs. Examples of inductive codes include additional stereotypes, life domains, issues of disclosure, and vulnerability factors. Two study members then applied this initial codebook to two transcripts and conducted line-by-line analysis. This process was repeated two more times until coder agreement was reached. The full coding team (five team members) used the refined codebook to manually code one transcript in Microsoft Word and conduct line-by-line comparison in group meetings. Differences in coding were discussed until agreement was reached. This process was repeated two more times until we developed a final codebook (S1 Table).

Using the finalized codebook, each IDI and FGD transcript was double coded in NVivo 14 [45,46]. Throughout coding, weekly group meetings were held to discuss data interpretation and coding challenges. The kappa statistic, calculated by NVivo, was used to measure interrater reliability, with kappa >0.7 indicating substantial agreement [47]. For transcripts with kappa <0.7, coding differences were resolved via line-by-line comparison in group meetings. All team members completed a summary memo after coding each transcript.

To examine stigma faced by ALWH and their experiences with suicidality, we began reviewing all code reports from ALWH and stakeholders. During this initial review, a central inductive theme related to premature mortality and death emerged, which informed the conceptualization of ‘death-centric stereotypes’ as a guiding idea. In this paper, *death-centric stereotypes* refer to pessimistic expectations about HIV survival or negative community beliefs regarding an ALWH’s presumed early mortality, and resulting abilities and potential due to their HIV status. Based on literature review and our prior experience working with this population, we theorized that death-centric stereotypes may be implicated in internalized stigma and suicidality. To better characterize the origin and impacts of death-centric stereotypes, we reviewed code reports for the following deductive codes: stereotypes, experienced stigma, internalized stigma, and suicidality. We conducted thematic analysis [48,49] to understand how these death-centric stereotypes influence experienced stigma, which together contribute to internalized stigma and suicidality. Throughout the analytic process, we drew upon key elements of The Health Stigma and Discrimination Framework [44], such as drivers, manifestations, and health impacts, to organize the four themes (death-centric stereotypes, experienced stigma, internalized stigma, and suicidality). The key themes are derived of data from the entire sample and are used to structure the analysis and focus the presentation of the results. As we explored these themes, we generated a model to depict the interplay between various forms of stigma and the impact on suicidality. *Experienced stigma* refers to actual experiences of discrimination against ALWH. *Internalized stigma* refers to the self-endorsement or acceptance of negative societal beliefs by ALWH themselves expressed through shame, low self-esteem or self-worth, guilt, and hopelessness [44].

#### Positionality.

Our data analysis team was comprised of five individuals with diverse cultural and academic backgrounds: a Greek American medical student pursuing a career in Psychiatry (Principal Investigator (PI)), an American research intern, a Malawian American PhD student in Nursing, and two Malawian RAs who conducted the interviews in Chichewa. All but one team member was based in Malawi during the time of the study. We recognize that our positionalities influenced data analysis and feel that the range of perspectives enriched our exploration of how HIV stigma impacts suicidality among ALWH. Malawian team members were instrumental in contextualizing stigma within local cultural frameworks, while non-Malawian team members contributed external perspectives informed by clinical and research backgrounds.

#### Inclusivity in global research.

Additional information regarding the ethical, cultural, and scientific considerations specific to inclusivity in global research is included in the Supporting Information (S3 Text).

## Results

### Participant characteristics

There were 68 total participants in this study.

We screened all ALWH aged 13–19 years presenting to five Teen Club clinic days. Of 118 ALWH screened, 26 ALWH (22%) screened positive for depression and 8 ALWH (6.78%) endorsed suicidal ideation.

Among 26 eligible participants, we enrolled 13 ALWH (5 females and 8 males) in this study. The mean age of ALWH was 16.3 years, with 6 participants in the 13–15 age group. A majority (n = 10) had only completed primary school. Under half (n = 6) had two living biological parents. Over half (n = 7) reported food and money insecurity in the past year. The mean BDI-II score was 18.3 (range 13–26). Females had a higher mean score of 19.6, compared to 17.5 in males. Two participants reported suicidal ideation on the BDI-II screener; one participant with active SI and the other with passive SI. One additional participant disclosed history of suicidal ideation during the interview. Nearly all participants (n = 11) self-reported mother-to-child transmission (MTCT) of HIV. One participant reported an accidental exposure (details unknown) as the mode of acquisition, and another was unsure of how they acquired HIV **(**Table 1).

**Table 1:** Characteristics of ALWH participants.

	Total ALWH (n = 13)	Female ALWH (n = 5)	Male ALWH (n = 8)
Age (years), mean(SD)	16.3 (2.1)	17.0 (2.4)	15.9 (2.0)
13–15, n(%)	6 (46)	2 (40)	4 (50)
16–19, n(%)	7 (54)	3 (60)	4 (50)
Education			
Primary, n(%)	10 (77)	4 (80)	6 (75)
Secondary, n(%)	3 (23)	1 (20)	2 (25)
Both Biological Parents Alive, n(%)	6 (46)	3 (60)	3 (38)
Past Year Food Insecurity, n(%)	7 (54)	3 (60)	4 (50)
Past Year Money Insecurity, n(%)	7 (54)	4 (80)	3 (38)
BDI-II^1^ score, mean(SD)	18.3 (3.5)	19.6 (1.5)	17.5 (4.1)
Suicidal Ideation, n(%)	3 (23)	1 (20)	2 (25)
BDI-II disclosure, n(%)	2 (15)	1 (20)	1 (13)
Interview disclosure, n(%)	1(8)	0	1(13)
Self-reported HIV Transmission			
MTCT^2^, n(%)	11 (85)	3 (60)	8 (100)
Accidental exposure, n(%)	1 (8)	1 (20)	0
Doesn’t know, n(%)	1 (8)	1 (20)	0

^1^Beck-Depression Inventory-II.

^2^Mother to Child Transmission.

Participant characteristics of each stakeholder group can be found in Table 2.

**Table 2:** Characteristics of stakeholder participants.

	Adolescent peers without HIV (n = 12)	School Teachers (n = 12)	Caregivers (n = 10)	HIV care providers (n = 12)	Mental health providers (n = 9)
Gender					
Female, n(%)	6 (50)	10 (83)	8 (80)	9 (75)	5 (56)
Male, n(%)	6 (50)	2 (17)	2 (20)	3 (25)	4 (44)
Age (years), mean(SD)	15.9 (2.2)	38.5 (13.4)	45.6 (15.5)	39.1 (7.7)	39.6 (6.9)
Education					
None, n(%)	0	0	0	1(8)	0
Primary, n(%)	6 (50)	0	6 (60)	0	0
Secondary, n(%)	6 (50)	0	4 (40)	2 (17)	5 (56)
Post-Secondary, n(%)	0	12 (100)	0	9 (75)	4 (44)

The mean age of adolescent peers without HIV was 16 years, and they had an even split by gender. Half of adolescent peers (n = 6) had completed primary school.

For schoolteachers (n = 12), the mean age was 39 years, and the majority were female (n = 10). All teachers had completed post-secondary education.

For caregivers (n = 10), the mean age was 46 years with the majority being female (n = 8). Over half (n = 6) had completed primary education, with the remaining (n = 4) having completed secondary education. Of note, nearly all caregivers reported food insecurity (n = 9) and money insecurity (n = 8) in the past year. Caregiver relations of ALWH included mother (n = 6), grandfather (n = 1), aunt (n = 1), uncle (n = 1), and older sister (n = 1). Caregiver occupations included business, guard, sales agent, and treatment supporter.

For HIV care providers (n = 12), the mean age was 29 years, and three-quarters were female (n = 9). All but one HIV care provider had completed secondary education (n = 2) or post-secondary education (n = 9). Occupations of HIV care providers included nurse managers, clinician, ART coordinator, midwife, data officer, and community health worker.

For mental health providers (n = 9), the mean age was 38 years and over half were female (n = 5). All participants had completed secondary (n = 5) or post-secondary (n = 4) education. Occupations of mental health providers included clinician, psychosocial counselor, and community health worker.

### Impact of HIV Stigma on Suicide Risk amongst ALWH

**Fig 1:**
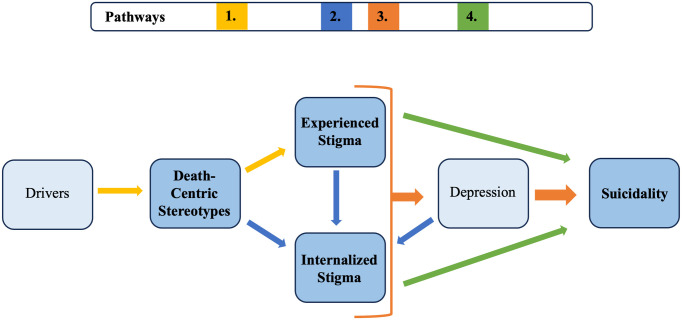
Stigmatization contributes to suicide risk for ALWH.

Fig 1 depicts pathways through which stigma contributes to suicide risk amongst ALWH. (1) Knowledge and beliefs (drivers) influence pervasive death-centric stereotypes, which contribute to experienced stigma and internalized stigma. (2) The development of internalized stigma is influenced by various factors, including stereotypes, experienced stigma, and depression. (3) Taken together, experienced and internalized stigma are contributory factors for depression, a known risk factor for suicide. (4) Both experienced stigma and internalized stigma can independently impact suicidal ideation and behaviors, producing a multifaceted, compounding effect for suicide risk.

In the Results section, we draw from broad structural elements of The Health Stigma and Discrimination Framework [44] to depict how death-centric stereotypes, together with experienced stigma, contribute to internalized stigma and suicide risk for ALWH.

*Of note, our findings support a significant impact from anticipated stigma, which is one of the major forms of stigma. Anticipated stigma had overlapping findings with experienced stigma, as anticipated stigma often results from witnessing stigma, so we do not delve into anticipated stigma in this analysis due to space constraints.*

### Part 1: Death-centric stereotypes

Participants cited discrete drivers of the death-centric stereotypes, particularly lack of knowledge about life expectancy on ARTs, and the stereotypical frail appearance of an individual with progressed HIV. Select participants, including adolescent peers, mental health counselors, and schoolteachers, cited an additional driver as the lasting impressions of the severe mortality toll during the early days of the AIDS epidemic in the 1990s. Additionally, participants recognized that HIV has no cure and thus believed HIV will eventually result in death. Participants seemed to place more emphasis on this belief, over the recognition of ARTs as a life-saving treatment.

In the following section, we discuss the three key death centric stereotypes which emerged as most relevant for ALWH: 1) HIV as a death sentence, 2) No future, and 3) Weak physical and mental capacity.

#### 1. HIV as a death sentences.

When participants were probed about community-held stereotypes about ALWH, the most common and immediate response from all participant groups, except HIV care providers, was “they are already dead.” This response reflects how deeply this stereotype is engrained in Malawian society. This belief, phrased in various ways (“dead person”, “finished,” “close to dying,” “corpse,” “moving around while already dead,” “time is running out”) speaks to the presumed imminent death of an ALWH.

This stereotype permeates into all facets of an ALWH’s life. In this example, a schoolteacher explained how school curriculums perpetuate this stereotype:

*“We have youth clubs in our schools where we sing some songs such as “AIDS is a killer, that’s why people die.” So, the students have that mindset that when one is exposed to HIV, they can die in a short period. That is what we teach them. We need to highlight that though such is the case, the healthcare providers provide counseling and guidance and when they follow that accordingly, they can live long.”* –female schoolteaher, age 30 years

These songs were initially created as public health awareness measures to educate adolescents on the health consequences of HIV and importance of HIV prevention. However, these songs have lost their relevance with the improved life expectancy for HIV. For a school-going ALWH, these songs continue to instil fear about their life expectancy and the curriculum reinforces stereotypes that are further spread through communities. This teacher noted that while she agrees that ALWH may die prematurely, counseling adolescents on the importance of ART adherence to prevent premature death could help to resist this tightly held belief that HIV is a death sentence.

#### 2. No future.

Grounded in the belief that HIV is a death sentence, a common public opinion was raised that ALWH have no future. This community perception is particularly relevant for schooling, employment, and family planning. In the example that follows, a participant recounted the common narrative about ALWH:

*“The belief that a lot of people use against a person with HIV is that a person with HIV cannot get educated, a person with HIV cannot work, a person with HIV cannot get married and that a person with HIV cannot do anything, even run a business.” – female ALWH, age 18 years, BDI 20*

In the following quote, a caregiver recounted a similar narrative:

***“****Some believe these adolescents cannot get married, have a future and even have children. People think their life is over.”* – female caregiver, age 39 years

The belief that ALWH have no future is particularly disheartening and disorienting for ALWH who are in a developmental life-stage with societal and familial pressures to begin planning for adult life. As heard from one ALWH participant with respect to schooling:

*“I have a lot of thoughts. Every child that goes to school thinks about what he or she wants to be. However, it’s not simple to achieve that; you must pass through a lot. So, it is difficult for a child like me who has HIV to go to school and work hard because of the stigma. This is so because when you are at school, you think about what they say about you. For instance, ‘What can a person with HIV, like you, achieve?’”* – male ALWH, age 15 years, BDI 15

In this quote, an ALWH described challenges to remain motivated to finish their schooling. The stereotype that ALWH have no future, may be internalized by ALWH, which creates feelings of self-doubt and uncertainty about their capabilities.

#### 3. Weak physical and mental capacity.

Participants often spoke of the stereotypical appearance of ALWH such as thin body habitus, hair thinning, rashes, and lightened skin. Furthermore, the public perceived an ALWH to be illness-prone and frequently coughing, reflecting their immunocompromised state. All participant groups noted that community members assume that all thin people have HIV, and presumptively distance themselves out of fear of HIV transmission. In addition, participants spoke about the belief that ARTs cause many side effects that can exacerbate their poor physical capacity. They specifically cited that ARTs cause general fatigue, sunken cheeks, and a weak or “crazy” mind.

In a focus group discussion, caregivers explained how adolescents are stigmatized based on their physical appearance:

*“Participant 1: Sometimes when the adolescent discloses HIV to his friends, they discriminate him because of the adolescent’s physical appearance. Sometimes the body reveals that this person really is on medication… The body has rashes, and some people develop sores…*

*Participant 2: When the adolescent is among friends, he or she might show some signs like being inactive and weak. So, when the friends see this, they discriminate him or her saying that he or she shouldn’t be among them."* – female caregivers, ages 47 and 50 years

This assumption of weak physical and mental capacity is linked to a discrete driver, specifically the frail appearance of a person with progressed HIV. This presentation is likely referring to AIDS wasting syndrome, which is associated with disease progression and death. Although this syndrome was more common prior to mass ART roll-out in Malawi, it has left an impression on current-day perceptions of HIV. All participant groups, except HIV care providers, explained that this stereotypical physical appearance is used as a justification to physically and socially distance themselves from ALWH, resulting in further stigmatization.

### Part 2: Experienced stigma

Participants across all groups reported that ALWH are subject to multiple forms of experienced stigma including gossip, insults and mockery, and physical and social distancing. In this section, we focus on four specific manifestations of experienced stigma: 1) Insults and mockery, 2) Reduced social and economic prospects for adulthood, 3) Caregiver lack of investment in schooling and other vital resources, and 4) Exclusion from community activities. These forms of experienced stigma appear to be driven by death-centric stereotypes and have serious consequences for ALWH’s future.

#### 1. Insults and mockery.

One of the main stigma experiences faced by ALWH was falling victim to mockery, with death-centric stereotypes commonly being employed as a direct insult. ALWH are subject to mockery primarily from peers and community members, but also from family members. In this example, an adolescent peer explained how parents may insult their ALWH after a wrongdoing:

*“Sometimes parents can be shouting at the adolescent because of a wrong act. The parents can say, ‘That’s why you are already dead, you have HIV.’ This depresses the adolescent.”* – female adolescent peer, age 19 years

Similarly, a caregiver explained how some caregivers may perpetuate the discrimination of their ALWH:

*“The real parents think the child is finished and is not important. There are some parents that discriminate a child and they pioneer the discrimination. Ordinary people copy from what the parents say and do the same. So, when an ordinary person says the same words that parents say, the child thinks he or she is not worthy.”* – male caregiver, age 22 years

These quotes exemplify harmful, but not uncommon situations, where caregivers spread death-centric stereotypes within and beyond family settings, which may contribute to depression. Because ALWH are directly told that they are already dead, they begin to believe these stereotypes, which contributes to the development of internalized stigma.

#### 2. Reduced social and economic prospects for adulthood.

The stereotype of HIV as a death sentence has immediate tangible impacts on marriage prospects and economic opportunities for ALWH. ALWH are told they cannot marry or have children, due to the fear of HIV transmission between partners and from mother to child. Many participants noted that people living with HIV are denied loans and employment opportunities, due to concern that the loan will not be repaid in the event of death.

In the quote below, an adolescent peer explained discrimination faced by people living with HIV:

*“According to what we learnt when I was in standard 5, they taught us that when a person with HIV wants to expand his or her business by getting a loan, people say, ‘Don’t give him or her a loan. He has HIV and can die soon.’ On the other hand, a person without HIV is given the loan. This gives the person with HIV many thoughts.”* – female adolescent peer, age 13 years

In this example, anticipated discrimination is believed to reduce economic prospects and livelihood, which may be particularly concerning in a setting where community members play a role in resource allocation for economic development. Although these examples are presented as cautionary tales, this adolescent peer reflected that these stories create “many thoughts”, or feelings of anxiety, about their future potential.

#### 3. Caregiver lack of investment in schooling and other vital resources.

Caregivers, HIV care providers, and mental health providers described how caregivers assume that their ALWH will not progress in life, so caregivers do not invest in their education or provide basic needs, like food. If money is scarce, caregivers may not pay school fees for ALWH, as it is considered a poor financial investment. This is especially relevant in a society like Malawi, where poverty is pervasive, and many families must make difficult decisions about finances.

*“I will talk about a scenario where in a family of four children, one of them is HIV positive. Most parents in this situation will automatically be selective. What happens is that, when resources are scarce, they don’t prioritize the resources on the child who is HIV positive. They feel that his or her future is uncertain because of the HIV status. This originates from the time the HIV pandemic had just been announced. It was so bad that people were just dying.”* – male psychosocial counselor, age 27 years

In this quote, a psychosocial counselor described how resources are allocated away from ALWH due to scarcity. This reality is further supported by a caregiver's report:

*“When my nephew was staying with his parents, he stopped school while in standard 2. Even though the mother was also on ART, the mother thought that the child had no future. I kept wondering why she was doing such things because her other two children were going to school. When granny asked my sister to at least request a transfer letter of the child so that the child should be enrolled at a school near granny’s residence, my sister refused saying that “I don’t have time to request a transfer letter for that child.” Granny had to use her own money to enroll the child in another school while her mother wasn’t involved in any way.”* – male caregiver, age 22 years

This lack of parental investment reinforces the community stereotype that ALWH do not have a future and fuels a cycle of marginalization, where ALWH are poorly positioned for future employment opportunities due to their limited education.

#### 4. Exclusion from community activities.

The public’s perception about their physical capacity fuels discriminatory practices where “sick looking people” may be excluded from certain activities, like playing football or participating in community development work. Community development work refers to activities for the betterment of the community, such as maintaining roads, digging drainage systems, and preparing food for funerals. Many of these activities require a level of physical exertion that is deemed by community members too taxing for ALWH, and ALWH are thus excluded.

In the example that follows, a mental health provider recounted the story of a patient who was excluded from playing football due to sores on his legs:

*“My colleague talked about a boy who had sores and when he went out to play, his friends were telling him that you are a sickling hence cannot play. With regards to this, if the adolescent’s talent was football, this would destroy his talent and the adolescent wouldn’t have the desire to go and play with friends, fearing that they will be insulting him by saying he is a sickling.”* – female mental health provider, age 39 years

In a similar example, an adolescent peer noted how an ALWH’s presumed weak physical capacity excludes them from community projects:

*“People start discriminating the person when it comes to various activities. For example, when it’s time to do community development works people say, ‘This person is already sick, we shouldn’t include him or her.’ …So, discrimination is really there, and the person isn’t involved in other activities because of his or her status.”* – male adolescent peer, age 18 years

Non-participation in community development works is particularly damaging in a community-oriented society like Malawi where individuals are expected to contribute to their community through physical labor. This exclusion from community activities prevents ALWH from feeling connected to fellow peers and community members, and sends a message that ALWH are not valuable to society, which reinforces low self-esteem.

### Part 3: Internalized stigma

The repeated exposure to experienced stigma, in addition to the death-centric stereotypes which dominate the rhetoric around ALWH, contribute to the development of internalized stigma. Participants described internalized stigma in many ways such as “feeling like a failure,” “unworthy,” and “hopeless.” From these discussions, we present three key manifestations of internalized stigma emerged that were directly linked to the death-centric stereotypes: 1) Low self-esteem, 2) Loss of future-oriented mindset, and 3) Self-Isolation.

#### 1. Low self-esteem.

“Feeling like a failure” was one of the most common ways that both ALWH and stakeholder participants described an ALWH’s perception of self. This overwhelming sense of “failing at life” reflects low self-esteem and a sense of worthlessness. Low self-esteem drives a negative perception of one’s importance in the world and ability to succeed in life.

In this example, a female caregiver explained how an ALWH internalizes stereotypes, which impacts her sense of self-worth:

*“I think they [negative community beliefs] can affect the adolescent since these issues are talked about everywhere. When the adolescent hears these things, she tries finding answers and starts blaming herself. She starts relating to the stories she hears and concludes that maybe she really is a failure and cannot achieve anything.”* – female caregiver, age 50 years

Stigma can make ALWH question their sense of belonging and value in society, which fuels self-doubt about their ability to progress through life and achieve their goals. As this participant pointed out, low self-esteem is also accompanied by self-blame, which was commonly cited by ALWH. Despite most participants reporting mother-to-child transmission of HIV, ALWH blamed themselves for being different than their peers and feeling like failures.

#### 2. Loss of future-oriented mindset.

After continuous exposure to external messaging that ALWH have no future, AWLH may internalize the belief that they themselves have no future, invoking a sense of hopelessness. The lack of belief in one’s own future disrupts personal planning for one’s future. A mental health provider noted that some ALWH are not interested in family planning, due to the belief that they will not bear children due to their HIV status. Many participants also reported that the presumed lack of future impedes an ALWH’s motivation for schooling and career.

This loss of future-oriented mindset is explained by two ALWH:

*“They believe that if someone has HIV, the person cannot do anything or even take care of a family… that’s the end of life. They believe the person cannot do anything because they feel the person is close to dying.”* – male ALWH, age 18 years, BDI 15

*“I feel like I cannot accomplish what I’m supposed to in the future. I want to become a bank manager but because I am stigmatized and insulted, I feel like I cannot be able to be what I want to be.”* – male ALWH, age 15 years, BDI 15

The first quote shows how external messaging that ALWH are close to dying is directly linked to having no future. This slowly wears down one’s confidence and ability to actualize one’s dreams. In the second quote, the ALWH reflected that he feels achieving his career goal is not possible due to the stigmatization he faces.

#### 3. Self-isolation.

Many participants also described self-isolating tendencies as a downstream consequence of stigma. It is important to note that self-isolation is both a symptom of depression and manifestation of internalized stigma. While it is not always possible to isolate the cause, both experienced stigma and stereotyping have a major influence on isolating tendencies, which can be devastating for an ALWH’s self-esteem.

A caregiver explained how this process develops over time:

*“They [negative community beliefs] can really push an adolescent backwards. This is so because when others have become aware of the adolescent’s [HIV] status, stigmatization starts. This brings depression and the person can no longer be free. As a result, the person cannot do other things, even school, freely because the person will just be thinking about it. Sometimes the person starts blaming himself or herself. This drags a person backwards. The person cannot even do any development works, as the person considers himself or herself not important and lost.”* –female caregiver, age 53 years

The participant noted that depression contributes to a lack of freedom, where an ALWH may experience shame in group settings, particularly out of fear of HIV status disclosure. Additionally, as previously explained in the results section, the stereotype that ALWH have weak physical capacity causes community members to exclude them from community development works. In this example, an ALWH exhibited internalized stigma and voluntarily disengaged from community works, due to a lack of self-efficacy and sense of belonging. Over time, self-isolation further intensifies depression, and feelings of hopelessness.

### Part 4: Suicidality

While there are many predisposing factors contributing to suicidality for ALWH, stigma is particularly salient in elevating suicide risk for ALWH. Stigma is a driver of depression, which is a known risk factor for suicide. However, participants’ stories revealed that experienced and internalized stigma can both independently compound suicide risk, without depression necessarily being a prerequisite.

Participants described various methods of suicidal behaviours, with intentional ART non-adherence standing out as an insidious and concerning practice. In addition, stigma perpetrated by family members seems to be particularly important in influencing suicide risk. The following section details 1) Intentional ART non-adherence, 2) Suicidal Behaviors, and 3) Role of Family.

#### 1. Intentional ART non-adherence.

One of the main methods of suicide discussed by participants was intentional ART non-adherence to allow for disease progression to AIDS and eventual death. Although ART non-adherence does not result in immediate death, this form of suicidal behavior is clinically concerning as a method of active suicidal ideation, wherein someone expresses a wish to die, and follows through with a plan.

An ALWH spoke about intentional ART non-adherence as a method of suicide:

*“I regard myself as a failure, so I don’t feel the need of taking medicine. When you have HIV, you ask yourself, “If I take this medicine, will I be healed? Will they stop mocking me?” So, you just stop taking medicine because you feel like you will not be able to play or chat with your friends even if you take the medicine. You feel like it’s better to just die.”* –male ALWH, age 15 years, BDI 15

In this example, the participant explained that because he has low self-esteem and knows that ARTs cannot cure HIV, he does not find value in taking his ARTs. In the face of stigmatization and resulting isolation, he feels his most favorable option is death, so he discontinues ARTs with the intent of eventual suicide by AIDS complications. Internalized stigma, marked by his sense of failure and preconceptions about his inability to socialize with friends, is likely playing a major role in his suicidality.

#### 2. Suicidal behaviors.

Participants also reported more traditional methods of suicide, particularly via intentional ART overdose, hanging, or ingesting poison.

In the story that follows, an ALWH described an argument with his elder brother in the home, resulting in physical abuse and his suicide attempt:

*“What I can remember is that my elder brother said that I am a dead person. He intentionally broke the pail because he was angry. I confronted him to stop behaving that way, and he stoned my head. I said, ‘If you want me dead you can just go ahead and kill me.’ He said I am already a dead person. So, I just went to the bedroom, opened my ART medications and as I was about to take them, my mother came and stopped me. Since he said that I am a dead person, I just wanted to kill myself.”* – male ALWH, age 15 years, BDI 20

This quote exemplifies how the stereotyping that ALWH are “already dead” can precipitate suicidality during periods of emotional crises. In this intimate story, a participant explained how an insult by his brother that he is “already dead” activated internalized thoughts. These thoughts caused him to question his worth in this world and consider suicide through ART overdose, which was averted by the intervention of his mother.

#### 3. Role of family.

Treatment by family members played a particularly important role in the stigmatization faced by ALWH, with potential for both positive and negative impacts. Stigmatization within the family home emerged as a particularly important risk factor for suicide. The three ALWH participants who shared details around their suicidality during the interviews, all also reported stigmatization within the home due to HIV, including abuse from family members.

The role of stigmatization from family members is highlighted by a mental health provider:

*“Not all the siblings take it so lightly. They isolate him saying that he is a corpse and cannot do any meaningful thing within their family. This led to depression and even suicide since the stigma is coming from within the family.” – male mental health provider (community health worker), age 49 years*

In this quote, a participant cited isolation and insults employing death-centric stereotypes from siblings as major factors for depression and suicide. This participant explicitly stated that stigmatization within the family poses a risk for suicide.

In contrast, supportive caregivers can help resist the stigmatization an ALWH faces:

*“*Ι *encourage the child I gave birth to so that the child should be independent and realize that one day, she is going to be a woman too... Love, motivation, and care is what is needed to help the child understand that HIV is not the end of life and 'I can achieve things in life.'"* – female caregiver, age 53 years

This ALWH’s mother shared the importance of reassuring her child that HIV is not a death sentence, and they can still have a productive future. Positive support from family members has the potential to decrease suicidality in ALWH who are otherwise hopeless about their future.

## Discussion

In this qualitative study, we investigated the impact of pervasive death-centric stereotypes and experienced stigma on the development of internalized stigma and suicidality, through IDIs with ALWH, and FGDs with adolescent peers, schoolteachers, caregivers, HIV care providers, and mental health providers. This study suggests that death-centric stereotypes are prevalent in the public discourse, including among family members, and positing that ALWH will die prematurely, which results in multiple forms of experienced stigma. ALWH face insults, reduced social and economic prospects for adulthood, caregiver lack of investment in school and basic needs, and exclusion from community activities. Stereotyping and experienced stigma contribute to internalized stigma, which is ultimately a risk factor for depression and suicide.

The public’s strong association of HIV with death underpins much of the stigmatization towards ALWH in Malawi. Despite substantial progress in improving HIV outcomes, study findings reveal that this pervasive fear of death deeply colors how the public perceives and speaks about ALWH. Based in the belief that HIV is a death sentence, ALWH are generally thought to die early, and thus are incapable of achieving their dreams. The stereotype that ALWH have no future has been identified in other countries in SSA. In Zimbabwe, ALWH spoke about the uncertainty of their future, which contributed to depression [50]. Multiple studies in Uganda identified presumed early mortality and poor health outcomes as common negative perceptions of ALWH [51,52]. Insulting ALWH by telling them directly that they are “already dead” has previously been described, including in Malawi [18,53]. ALWH are both denied vital resources, such as food and education, and have reduced prospects of achieving important adult milestones. This caregiver denial of basic needs, justified by the belief that ALWH will soon die, is well cited in the literature [51–53]. Reduced support for education is particularly concerning as it limits scholastic and employment opportunities, and may devalue an ALWH’s sense of self-worth. Further, lack of education exacerbates internalized stigma, as lower levels of educational achievement are associated with higher levels of internalized stigma [31]. The impact of these stereotypes can be detrimental for ALWH who are in a critical period marked by identity formation and planning for one’s future [54].

This study found that death-centric stereotypes and experienced stigma contribute to clear manifestations of internalized stigma, including low self-esteem, loss of future-oriented mindset, and self-isolation. Low self-esteem is common amongst ALWH [55,56]; it drives self-doubt and negatively impacts their ability to plan for the future and actualize their dreams. As these ALWH progress through life, social distancing enacted by others ultimately results in ALWH isolating themselves [51]. The self-isolation is driven by shame and fear of status disclosure in group settings [57], and may be exacerbated by feelings of otherness and unworthiness. Both isolation from others and self-isolation may contribute to a “social death,” wherein an ALWH is treated as dead, or non-existent, and is denied community roles [58]. People living with HIV may additionally be predisposed to a social death due to the public’s association between HIV and death [58,59]. These factors taken together, compounded by the intense need for belonging in adolescence, may cause ALWH to lose their sense of purpose, thus contributing to increased suicide risk.

When ALWH are repeatedly reminded of their presumed premature death through others’ words and actions, ALWH may lose their will to live. This study suggests that the social devaluation resulting from one’s health condition prevents ALWH from meaningfully preparing for adulthood, including completing school, establishing employment, and pursuing marriage. ALWH who internalize that HIV will cause them to die prematurely and who face mockery, abuse, and isolation, may prefer to end their life by suicide. The most commonly discussed suicide method was ART non-adherence, with the intention of eventual death from AIDS. While the elevated risk of suicide amongst ALWH is well-cited in the literature [18,60–63], there is less recognition of ART non-adherence as a suicide method; it is rather understood as poor HIV care engagement. Traditional suicide methods, such as overdose, hanging, and ingesting poison, require decisive action to be taken by the individual resulting in quick death. In contrast, slow suicide [50] via ART non-adherence actually requires less effort by the ALWH. Additionally, in some cases, ART non-adherence may be further motivated by stigma reduction and the desire for social inclusion. As previously reported, by stopping ARTs, ALWH can temporarily "remove" this physical marker of HIV, and fit in with their peers [19]. The elevated risk of suicide amongst ALWH necessitates the development of suicide prevention programming that is particularly attentive to insidious methods of suicidal behavior, notably ART non-adherence.

While there are many contributing factors to suicidal behavior amongst ALWH, severe stigmatization from family, particularly acts of abuse, appears to be strongly associated with suicidality, based on the discussions with all participant groups. Participants discussed various forms of physical, verbal, and psychological abuse, such as insults, stoning, isolation, and withholding of basic needs. ALWH with deceased parents were most vulnerable, as this abuse was often perpetrated by stepparents and replicated by siblings. This association between familial abuse and suicidal ideation aligns with previous studies in SSA [18,53,61]. Although adolescents begin to detach from family and identify more with peers during these developmental years [54], adolescents are still dependent upon family for basic needs and social support. The role of family in perpetuating stigma and contributing to ALWH’s suicidal ideation highlights the need to identify and protect vulnerable ALWH residing in unsafe homes, and address interpersonal stigma within home settings in stigma reduction and mental health programming for ALWH.

### Recommendations

The findings from this study identify two areas for critical intervention in the care of ALWH: internalized stigma and suicide prevention. Internalized stigma is a critical area for intervention due to its strong association with depression and suicide [16,24–26,60,64]. While little is known about how to address internalized stigma, particularly amongst ALWH in LMICs, a systematic review identified ART provision, social empowerment, economic strengthening, and cognitive-behavioral therapy as promising interventions [65]. Given that cognitive-behavioral therapy is also an effective treatment for depression, this tool should be leveraged in the development of stigma-reduction interventions. Stigma-reduction interventions should help ALWH resist negative stereotypes and dispel myths around early mortality, as death-centric stereotypes are clear drivers of internalized stigma. Further, interventions should be attentive to other factors, such as discrimination in schools and abuse at home, which contribute to internalized stigma [24,66]. Notably, this study identified school curriculums as a potential area where stigmatization is perpetuated against ALWH. As such, school leaders and Ministry of Education officials should be appropriately engaged to revise the school curriculum, which works towards a multi-faceted stigma-reduction approach.

Beyond addressing stigma, there needs to be a prioritization of resources towards suicide prevention, screening, and management. This requires developing locally adapted and translated tools for suicide screening, bolstering the availability of counselors to manage patients with suicidal ideation, and escalating care where appropriate. Suicide assessment for ALWH should be particularly attentive to the motivation behind ART non-adherence, as this study identified non-adherence as a common method of suicide. Suicide prevention will require closely engaging caregivers to halt the perpetuation of stereotypes, through education about ALWH life expectancy on ARTs and encouraging resource investment for their ALWH. Lastly, suicide is still criminalized in Malawi, which may perpetuate stigma and discourage help-seeking behavior, thus hampering suicide prevention efforts [23]. Involving policy makers and advocating for the decriminalization of suicide will allow for meaningful change on a systemic level.

### Limitations

A few limitations of this qualitative study should be noted. First, the ALWH participants were purposively sampled from a population of ALWH engaged in HIV care specifically tailored for their age group, which limits the generalizability of these findings to ALWH accessing care in the general HIV ward or ALWH not engaged in any HIV care. However, we might expect many ALWH in Malawi to face similar cultural manifestations of stigma, so these results still provide important insights. Second, the interview guides did not specifically ask about experiences of internalized stigma or suicide, so there may be elements of these topics that were not fully investigated. However, the interviews revealed rich participant experiences with data saturation being reached, so this data is still valuable in furthering our understanding of these critical issues. Additionally, this was a small exploratory study, and we acknowledge the proposed pathways in Fig 1 are complex. Additional research is needed to further interrogate these pathways. Lastly, if we want to effectively tackle suicide prevention on multiple levels, we must address the criminalization of suicide, which requires interviewing key stakeholders such as policy makers and law enforcement. This study did not interview such stakeholders but instead included key perspectives from caregivers, teachers, adolescent peers, and healthcare providers.

## Conclusions

This qualitative study revealed how death-centric stereotypes influence distinct manifestations of experienced stigma, which contribute to the development of internalized stigma and suicidality amongst ALWH in Malawi. Identified death-centric stereotypes include HIV as a death sentence, and the beliefs that ALWH have no future, and weak physical and mental capacity. Theses stereotypes form the grounds for experienced stigma, including direct insults, reduced economic and social prospects adulthood, parental lack of investment in education and other vital resources, and exclusion from community activities. Manifestations of internalized stigma include low self-esteem, loss of future-oriented mindset, and self-isolation. These stigmata, compounded by the high prevalence of depression, amounts to an elevated risk of suicide for ALWH, particularly though intentional ART non-adherence. Notably, family members' treatment can influence suicide risk, exerting either protective or harmul effects. These findings highlight the need to develop multi-level stigma reduction interventions to address internalized stigma and halt the perpetuation of death-centric stereotypes, particularly within the home. In addition, suicide screening and prevention programming is urgently needed for ALWH as the stigma attached to their health condition places ALWH at increased risk of suicide.

## Supporting information

S1 Text IDI guide. (DOCX)

S2 Text FGD guide. (DOCX)

S3 Text Inclusivity in global research. (DOCX)

S1 Table Codebook. (DOCX)
