# Supplementary material for: ‘You feel like it’s better to just die’: Death-centric stereotypes and stigma contribute to suicide risk for adolescents living with HIV in Malawi
Source: PLOS Glob Public Health. 2025 Dec 29;5(12):e0005655. doi: 10.1371/journal.pgph.0005655 (PMC12747334; doi:10.1371/journal.pgph.0005655)
Supplement: S1 Text — (DOCX) [file pgph.0005655.s001.docx]

**In-Depth Interview (IDI) Guide for Adolescents Living with HIV (ALWH)**

Strategies for Adolescent Reduction of Stigma (STARS) Study

*Note: This guide was changed during data collection to improve participant understanding and to better align questions with study goals. The content remained largely the same, however the wording of some questions differed. Guides were changed in response to data review and at the suggestion of research assistants who conducted the interviews.*

Hello, I am working with researchers at UNC Project-Malawi. Thank you for consenting to participate in this interview about your experience with HIV, depression, and stigma.

I will be leading this interview with my colleague. We will also be recording the interview so we can refer to the conversation in the future. We are hopeful that our conversation will help us offer better healthcare for other adolescents like you. There are no right or wrong answers. What is discussed in this interview will remain private. Any information we use will be de-identified, meaning that no one will be able to know that you were talking with us about these issues. Some of the topics we discuss may be sensitive or bring up difficult memories, so please let us know if you want to take a break or end the interview early.

Before we begin, I would like to ask if you could turn off your phone.

Any questions before we get started?

**Ice Breaker**

1. Can you tell me a little bit about what a typical day looks like for you? What about on the weekends? What activities do you enjoy?
2. Who are the most important people your life?

- Probe: Anyone else?

1. Now thinking back to the previous question, what ‘roles’ do you take on in your community or family? For example, friend, student, son/daughter, brother/sister, football player, church member, employee, girlfriend/boyfriend

In order of importance, which of these 3 roles matter the most to you?

Interviewer, take note:

1) ________________ 2) ________________ 3) ________________

**Stigma Intro**

Now, I’d like to focus the discussion on stigma.

1. Have you heard of this term stigma? How would you describe it in your own words?

I’d like to share a definition. Stigma is the negative ways people are viewed or treated because of their conditions. When someone is treated unfairly because of a difference, this is discrimination, which often originates from stigma.

1. Do you agree with this definition? What would you change?

**HIV Stigma**

HIV is a difference that is often associated with stigma. I would like to start with a story.

*Thandi is a 14-year-old living with HIV. Thandi needed help with her school assignments after being sick, so she explained her situation to her classmate and disclosed her HIV status. In the weeks that followed, Thandi’s classmate stopped eating lunch and playing with her. Thandi now feels embarrassed about her status and is scared that her classmate will spread gossip to other students in her class.*

1. What do you think about what is happening to Thandi?
2. How do other people react when they find out an adolescent has HIV?

- Probe: What about parents? What about siblings? What about friends? What about healthcare providers?

1. What are some common negative beliefs, if any, that others hold about adolescents living with HIV?

- Probe: Do you agree?
  - IF YES - Probe: Has anyone ever said these things about you? Or do you believe this about yourself?
  - IF NO- Probe: How are you able to resist this stereotype? What makes you strong?
- Probe: Is this a common belief in your community? If so, why do you think people believe this?

[Interviewer: please continue with these, if they have not already been stated. Probe with each statement below.]

Here are some other common beliefs about people living with HIV.

- - People don’t want to date someone with HIV
  - People believe that someone with HIV probably uses drugs.
  - People believe that someone with HIV is sexually promiscuous.
  - People believe that a person acquired HIV as a punishment for something they did wrong.
- Probe: Do you agree?
  - IF YES - Probe: Has anyone ever said these things about you? Or do you believe this about yourself?
  - IF NO- Probe: How are you able to resist this stereotype? What makes you strong?
- Probe: Is this a common belief in your community? If so, why do you think people believe this?

1. Some people experience stigma around their HIV diagnosis. What has been your personal experience with HIV stigma? Have you ever been judged or treated negatively due to your HIV status?

- Probe: If you feel comfortable, please share some examples.

[Interviewer, encourage them to share a story]

- Probe: What about in school? At home? At church?

1. Now, think back to the 3 roles you identified that were most important to you:

(For example, being a good student? Son or daughter? Sibling? Friend? Other?)

You said, 1)______________ 2) ________________ 3)________________

Note to interviewer: probe each role with these questions:

- What things must you do in order to be a good _______ ?
- How does HIV stigma, if at all, change the way in which you are able to do the activities that are required of a good _______?

1. Let’s think about an adolescent with HIV who is a good _______ (student, for example) compared to an adolescent with HIV who is a bad ________. Do people think better of this adolescent because they are a good ______, even though they know they have HIV? Why or why not?

- Probe: Does being a good _____ decrease the stigma others might feel towards them? Why or why not?

1. How does stigma impact taking your ART medications, if at all? What about going to ART appointments?
2. How does stigma from HIV, if at all, influence the way you view yourself?
3. What do you do to cope with the stigma from HIV?

- Probe: What else could other people do to help you cope? What about healthcare providers?

**Depression Stigma**

Now, we are going to change topics a little and talk about depression. I would like to share a story.

*Mphatso is a 17-year-old boy. After his father passed away 1 year ago, he became very sad, quiet, and hopeless about his future. His grades dropped and he stopped playing football with his friends. His friends stopped inviting him to play together because they are concerned he may cause issues in the group, as he is always sad and lonely.*

1. What do you think about what is happening to Mphatso?
2. Have you heard of what depression is? Could you describe it in your own words?

I’d like to share a definition again. Depression is a condition where someone feels sad and overwhelmed, feels hopeless about their future, and no longer enjoy activities they normally do. This person may also have changes in their sleeping and eating habits. They may have difficulty concentrating. They may even have thoughts of harming themselves. Depression usually lasts at least 2 weeks but may last much longer.

Similar to HIV, depression often has stigma associated with it.

1. What do other people think about an adolescent who is depressed? What are some negative beliefs, if any, that people believe?

- Probe: Do you agree?
  - IF YES - Probe: Has anyone ever said these things about you? Or do you believe this about yourself?
  - IF NO- Probe: How are you able to resist this stereotype? What makes you strong?
- Probe: Is this a common belief in your community? If so, why do you think people believe this?

[Interviewer: please continue with these, if they have not already been stated. Probe with each statement below.]

Here are some other common beliefs about people with depression

- 1. People with depression are dangerous and unpredictable.
  2. People think that having depression is damaging to your reputation.
  3. People think that having depression makes you weak.
- Probe: Do you agree?
  - IF YES - Probe: Has anyone ever said these things about you? Or do you believe this about yourself?
  - IF NO- Probe: How are you able to resist this stereotype? What makes you strong?
- Probe: Is this a common belief in your community? If so, why do you think people believe this?

1. From the screening for the study, it shows that you have been experiencing signs of depression. Can you tell me more about this? Can you share with me what symptoms you are experiencing?
2. You just told me you were experiencing these symptoms of depression. How have people treated you differently because you were experiencing these symptoms?

- Probe: If you feel comfortable, please share some examples.

[Interviewer, encourage them to share a story]

- Probe: What about in school? At home? At church?

1. Now, remember those three roles that are important to you?

You said, 1)______________ 2) ________________ 3)________________

- Probe for each role:
  - What activities must you do to be a good _______?
  - How does stigma from depression, if at all, impact your ability to do these things required of a good _______ ?

1. Let’s think about an adolescent with depression who is a good _______ (student, for example) compared to an adolescent with depression who is a bad ________. Do people think better of this adolescent because they are a good ______ , even though they are depressed? Why or why not?

- Probe: Does being a good _____ decrease the stigma others might feel towards them? Why or why not?

1. What do you do to cope with the stigma from depression?

- Probe: What else could others do to help you cope? What about healthcare providers?

**Intersectional Stigma**

Research has shown that HIV and depression are conditions that often occur together.

1. How do you think that the stigma from HIV, if at all, impacts your mental health?
2. Do you think the experience of HIV stigma is different than the experience of depression stigma? If yes, how so? Is one worse than the other?
3. I’d like to understand more about what happens when people feel stigma from both HIV and depression. Can you imagine what someone would feel like if they have both HIV and depression?

- PROBE: What is it like for you?
  - Interviewer, please encourage the participant to share a story here. For example, if they say “I feel like a failure.” Probe: Can you think back to a specific moment when you felt like a failure? Can you tell me the story?

[Interviewer- if participant states they do not feel stigma from depression, follow with questions below.]

- Probe: Even though you don’t feel stigma from depression, how might you think someone else would experience stigma from both HIV and depression?
- Probe: You can think back to Thandi and Mphatso. What if one person faced the stigma that both Thandi and Mphatso face?

1. What helps you cope with the stigma you feel from depression and HIV?
2. Why do you think some people are better able to manage the stigma than others? What makes some people stronger in dealing with the stigma?
3. What can we do to help an adolescent with both depression and HIV?
4. Do you have anything else you would like to add?

This is the end of our interview. Thank you for your participation.
