# Supplementary material for: ‘You feel like it’s better to just die’: Death-centric stereotypes and stigma contribute to suicide risk for adolescents living with HIV in Malawi
Source: PLOS Glob Public Health. 2025 Dec 29;5(12):e0005655. doi: 10.1371/journal.pgph.0005655 (PMC12747334; doi:10.1371/journal.pgph.0005655)
Supplement: S2 Text — (DOCX) [file pgph.0005655.s002.docx]

**Focus Group Discussion (FGD) Guide for Stakeholders**

Strategies for Adolescent Reduction of Stigma (STARS) Study

*Note: This guide was changed during data collection to improve participant understanding and to better align questions with study goals. The content remained largely the same, however the wording of some questions differed. Guides were changed in response to data review and at the suggestion of research assistants who conducted the interviews.*

*The guide was also slightly adapted based on focus group population (adolescent peers without HIV, caregivers of ALWH, schoolteacher, mental health counselors, and government personnel). The main questions remained the same, but some questions were specified based on the participant’s relationship to an adolescent living with HIV and depression. Specific adaptations are indicated in questions 5, 12, and 18.*

Hello, I am working with researchers at UNC Project-Malawi. Thank you for consenting to participate in this focus group about HIV, depression, and stigma. I will be leading this interview with my colleague. We will also be recording the interview so we can refer to the conversation in the future. We are hopeful that our conversation will help us offer better healthcare for adolescents living with HIV and depression. There are no right or wrong answers. What is discussed in this interview will remain private. Any information we use will be de-identified. Some of the topics we discuss may be sensitive, so please let us know if you want to take a break or leave the discussion.

Before we begin, I would like to ask if you could turn off your phone.

Any questions before we get started?

**Icebreaker**

Interviewer: please assign numbers to participants. Ask participants to reference their number before speaking.

**Introduction**

1. Have you heard of the term stigma? How would you describe it in your own words?

I’d like to share a definition. Stigma is the negative ways people are viewed or treated because of their conditions. When someone is treated unfairly because of a difference, this is discrimination, which often originates from stigma.

1. Do you agree with this definition? What would you change or add?

**HIV Stigma**

HIV is a condition that is often associated with stigma. I would like to start with a story.

*Thandi is a 14-year-old living with HIV. Thandi needed help with her school assigments after being sick, so she explained her situation to her classmate and disclosed her HIV status. In the weeks that followed, Thandi’s classmate stopped eating lunch and playing with her. Thandi now feels embarrassed about her status and is scared that her classmate will spread gossip to other students in her class.*

1. What do you think about what is happening to Thandi?
2. How do people react when they find out an adolescent has HIV?

- Probe: What about parents? What about siblings? What about friends? What about healthcare providers?

1. Sometimes adolescents living with HIV are treated differently or unfairly because of their HIV status. Could you tell us about that? Could you share any stories?

- Prompts specified by group:
  - *For adolescent peers*: Do you know anyone your age with HIV? How do they get treated differently in school because of this? What about outside of school, for example in sports or in the neighborhood or in church?
  - *For schoolteachers:* Do you know of any students with HIV in your classes? **But as a reminder, we do not want to know names.** How do they get treated differently in school? Have you seen any bullying (verbal or physical mistreatment by a peer)?
    - *Probe:* If you do not know any students with HIV, what stories have you heard?
  - *For caregivers of ALWH*: How is your family member treated differently due to their HIV status? In what environments does this come up?
  - *For mental health counselors*: How do you feel like your patient is ever treated differently due their HIV status? Do they bring up these issues in appointments with you? What other things have you noticed working with adolescents living with HIV?
  - *For government personnel/clinic leaders:* What are larger community impressions of adolescents living with HIV? Do these impressions of ALWH persist amongst clinic leaders like yourself?
    - If you see patients, do they discuss HIV stigma in appointments with you?

1. What are some common negative beliefs, if any, that people have about adolescents living with HIV?

- Probe: Is this a common belief in your community? If so, why do you think people believe this?

[Interviewer: please continue with these, if they have not already been stated]

Here are some other common beliefs about people living with HIV.

- - People believe that someone with HIV is sexually promiscuous
  - People believe that a person acquired HIV as a punishment for something they did wrong.
- Probe: Is this a common belief in your community? If so, why do you think people believe this?

1. How does HIV stigma, if at all, impact an adolescent’s ability to participate in important life activities? (such as performing well in school, playing sports, completing household chores)
2. Let’s think about an adolescent with HIV who is a good student, for example, compared to an adolescent with HIV who is a bad student. Do people think better of this adolescent because they are a good student, even though they have HIV? **Why or why not?**

- Probe: Does being a good student decrease the stigma others might feel towards them? **Why or why not?**

**Depression**

Now, we are going to change topics a little and talk specifically about depression. I would like to share a story.

*Mphatso is a 17-year-old boy. After his father passed away 1 year ago, he became very sad, quiet, and hopeless about his future. His grades dropped and he stopped playing football with his friends. Even though his friends do not know his status, they stopped inviting him to play together because they are concerned he may cause issues in the group, as he is always sad and lonely.*

1. What do you think about what is happening to Mphatso?
2. Have you heard of what depression is? How would you describe it?

Here is one way that people have described depression. Depression is a condition where someone feels sad and overwhelmed, feels hopeless about their future, and no longer enjoy activities they normally do. This person may also have changes in their sleeping and eating habits. They may have difficulty concentrating. They may even have thoughts of suicide and wanting to harm themselves. Depression usually lasts at least 2 weeks but may last much longer.

Similar to HIV, depression often has stigma associated with it.

1. FOR CAREGIVERS: All of you were invited to participate in this focus group because your adolescent screened positive for depression.

In Malawi, how do parents respond if an adolescent is diagnosed with depression?

- Probe: How much of a problem, if any, do you think suicide (or thoughts of wanting to kill oneself) is among youth in your community?
- Probe: How would a parent be treated if their child was depressed or tried to commit suicide?

1. Could you tell us about how adolescents with depression might be treated differently?

- Prompts specified by group:
  - *For adolescent peers*: Do you know anyone your age with depression? How do they get treated differently, if at all, because of this? For example in school, sports, or in the neighborhood?
  - *For schoolteachers:* Have you seen any signs or symptoms of depression in any of your students? How have you noticed that they get treated differently by their peers in school?
  - *For caregivers of ALWH*: How do you feel like adolescents (like your child) are treated differently, if at all, because they are depressed? In what environments does this come up?
  - *For mental health counselors*: How do you think your adolescent patient is stigmatized due to having depression? What sorts of things do they say to you?
  - *For government personnel/clinic leaders:* How do you think an adolescent is stigmatized due to having depression? What are broader community impressions of adolescents with depression?

1. What are some common negative beliefs that people have about adolescents who are depressed?

- Probe: Probe: Is this a common belief in your community? If so, why do you think people believe this?

[Interviewer: please continue with these, if they have not already been stated]

Here are some other common beliefs about people who are depressed.

- - People with mental illness are dangerous and unpredictable.
  - People think that having depression makes you weak.
- Probe: Probe: Is this a common belief in your community? If so, why do you think people believe this?

1. For adolescents with depression, how does the stigma from depression, if at all, impact their ability to participate in important life activities? (such as performing well in school, playing sports, performing household chores)
2. Let’s think about an adolescent with depression who is a good student, for example, compared to an adolescent with depression who is a bad student. Do people think better of this adolescent because they are a good student , even though they are depressed? **Why or why not?**

- Probe: Does being a good student decrease the stigma others might feel towards them? **Why or why not?**

**Intersectional Stigma**

Research has shown that HIV and depression are conditions that often occur together. Experiencing stigma from multiple conditions is called intersectional stigma.

1. I’d like to understand more about stigma faced by adolescents with both HIV AND depression. What do you think of them?

- Probe: What particular challenges, if any, do you think they face?
- Probe: How is this different than only facing stigma from depression OR HIV?

1. Why do you think some adolescents with HIV & depression are more resilient than others? What are their positive coping mechanisms?
2. How much or how little of a problem do you think stigma of HIV and depression is a problem in our community? How do you think we can help?

- Prompts specified by group:
  - *For adolescent peers*: What do you think could help in decreasing stigma faced by your peers with HIV and depression?
  - *For schoolteachers:* What do you think could help in decreasing stigma faced by adolescents with HIV and depression? Any school policies or campaigns?
  - *For caregivers of ALWH*: What do you think could help in decreasing stigma faced by family members like yours with HIV and depression?
  - *For mental health counselors*: What do you think could help in decreasing stigma faced by adolescents with HIV and depression? From a counseling perspective or otherwise? Policies?
  - *For government personnel:* What do you think could help in decreasing stigma faced by adolescents with HIV and depression? Any changes on a clinic level or policy changes?

1. Do you have anything else to add?

This is the end of our focus group. Thank you for your participation.
