# Supplementary material for: ‘You feel like it’s better to just die’: Death-centric stereotypes and stigma contribute to suicide risk for adolescents living with HIV in Malawi
Source: PLOS Glob Public Health. 2025 Dec 29;5(12):e0005655. doi: 10.1371/journal.pgph.0005655 (PMC12747334; doi:10.1371/journal.pgph.0005655)
Supplement: S1 Table — (DOCX) [file pgph.0005655.s004.docx]

Note: This document contains the complete codebook for the STARS study, however the analysis only draws from select codes.

| **Theme** | **Subtheme** | **Code** | **Code definition** | **Code type** |
| --- | --- | --- | --- | --- |
| **What Matters Most (WMM)** | Routine life | Daily activities | Use this code when adolescents living with HIV (ALWH) describe their daily activities. This is question #1 on the in-depth interview (IDI) guide. | Deductive |
|  |  | Important people | Use this code when ALWH name important people/figures in their life. This is question #2 on the IDI guide. | Deductive |
|  |  | Roles | Use this code when ALWH describe their roles and responsibilities. This is question #3 on the IDI guide. | Deductive |
|  | HIV stigma (HIVS) | HIVS impedes WMM | Use this code when talking about how HIVS impedes an ALWH's ability to participate in activities that matter most to them (school, church, etc). This is specifically asked in Q10 on IDI guide and Q7 on focus group discussion (FGD) guide, however it may come up in other places in the interview. | Deductive |
|  |  | Roles protect against HIVS | Use this code when talking about how an ALWH fulfilling roles protects against further stigmatization from HIV. This is specifically asked in Q11 on IDI guide and Q8 on FGD guide, however it may come up in other places in the interview. | Deductive |
|  |  | Roles do not protect against HIVS | Use this code if a participant says that fulfilling roles does NOT protect against HIV stigma. | Deductive |
|  | Depression stigma (DS) | DS impedes WMM | Use this code when talking about how *depression stigma* impedes an ALWH's ability to participate in activities that matter most to them (school, church, etc). This is specifically asked in Q20 on IDI guide and Q14 on FGD guide, however it may come up in other places in the interview. | Deductive |
|  |  | Roles protect against DS | Use this code when talking about how an ALWH fulfilling roles protects against further stigmatization from depression. This is specifically asked in Q21 on IDI guide and Q15 on FGD guide, however it may come up in other places in the interview. | Deductive |
|  |  | Roles do not protect against DS | Use this code if a participant says that fulfilling roles does NOT protect against depression stigma. | Deductive |

| **Theme** | **Subtheme** | **Code** | **Code definition** | **Code type** |
| --- | --- | --- | --- | --- |
| **HIV** | General | Understanding of stigma | Use this code when participants explain what stigma means to them. | Deductive |
|  |  | Thandi responses | Use this code when participants have rich responses to the Thandi vignette. | Deductive |
|  |  | Accurate knowledge | Use this code when a participant demonstrates accurate knowledge about how HIV is spread, treated, etc. | Inductive |
|  |  | Changes over time | Use this code to describe changes in perceptions of HIV stigma over time. | Inductive |
|  |  | No HIVS | Use this code if participants say they have not experienced HIV stigma. If stated, please include why they have not experienced stigma (it is often due to keeping HIV status secret). | Inductive |
|  |  | Vulnerability factors | Use this code when participants describe other social determinants of health that may influence how HIV diagnosis is experienced. | Inductive |
|  |  |  |  |  |
|  | Settings |  | Use this code for stigma that occurs in discrete settings not otherwise included in the list below | Inductive |
|  |  | Home | Use this code to describe stigma that occurs specifically in home settings and within the family unit. This applies for parents, stepparents, and siblings, or other people residing in the home. | Inductive |
|  |  | Community | Use this code for stigma that occurs in community settings, or for broad/unspecified community perceptions of ALWH. Community settings include church, sports, market, and other village settings. | Inductive |
|  |  | School | Use this code to describe stigma that occurs in school settings. This can include from teachers and other peers. | Inductive |
|  |  | Healthcare | Use this code to describe stigma that occurs in healthcare settings like the clinic or hospital. This can refer to stigma from healthcare workers, other patients, or how the built environment facilitates stigma. | Inductive |

|  | HIVS drivers |  | Use this code for drivers of HIVS that is not included in one of the below codes | Inductive |
| --- | --- | --- | --- | --- |
|  |  | Fear of HIV transmission | Use this code when talking about behaviors that drive HIV stigma due to fear of HIV transmission. | Deductive |
|  |  | Fear of ARTs | Use this code when participants describe a fear of ART medications themselves. | Deductive |
|  |  | Inaccurate knowledge | Use this code when someone describes inaccurate knowledge about how HIV is spread, treated, etc. | Deductive |
|  | HIVS stereotypes |  | Use this code for discussion around stereotypes and prejudices of ALWH. Use this code if it does not fit into one of the categories below. | Inductive |
|  |  | Weak or sickly | Use this code for the stereotype that an ALWH is weak, sickly, or illness prone. | Deductive |
|  |  | Death sentence | Use this code when participants say that HIV is a death sentence or may cause a premature death. | Deductive |
|  |  | No future | Use this code when participants describe a lack of future or an inability to achieve dreams or contribute to society due to HIV. | Deductive |
|  |  | Punishment or curse | Use this code when participants describe HIV as a punishment for wrongdoing (such a promiscuity) or a curse. | Deductive |
|  |  | Uses substances | Use this code for the stereotype that ALWH uses drugs OR alcohol. | Inductive |
|  |  | Decreased marriage potential | Use this code for the stereotype that ALWH have decreased marriage OR dating potential. Also, use this code also for references of HIV's presumed impact on romantic relationships. | Inductive |
|  |  | Promiscuous | Use this code when participants say ALWH are promiscuous. | Deductive |
|  |  | Government beneficiaries | Use this code when participants say ALWH are government beneficiaries. | Inductive |
|  | Experienced stigma |  | Use this code to describe experienced, witnessed, or anticipated stigma that does not fit into one of the discrete categories below. | Inductive |
|  |  | Gossip | Use this code to describe gossip as a manifestation of HIVS. | Deductive |
|  |  | Insults | Use this code to describe insults and mocking as a manifestation of HIVS. | Deductive |
|  |  | Distancing | Use this code to describe someone's physical distancing from an ALWH (for example not sharing cups or playing football etc). | Deductive |
|  | Internalized stigma |  | Use this code to describe self-stigma, or negative opinions of self, that results from other manifestations of HIV stigma. | Deductive |
|  | Associated stigma |  | Use this code to describe stigma experienced due to association with an ALWH. | Deductive |
|  |  |  |  |  |
|  | Negative consequences |  | Use this code for negative consequences not otherwise listed below. | Inductive |
|  |  | Decreased HIV care engagement | Use this code to describe decreased HIV care engagement resulting from HIV stigma. | Deductive |
|  |  | Isolation | Use this code to describe pervasive physical/social isolation due to HIV. This can be self-isolation or public's isolation but is more severe than simply physical distancing. | Inductive |
|  |  | Hopelessness | Use this code to describe hopelessness around one's future. Include passive suicide "it's better to die" under this code. | Inductive |
|  |  | Poor mental health | Use this code to describe worsened mental health resulting from HIV stigma. This includes depression, anxiety, unhealthy substance use. | Deductive |
|  |  | Suicidality | Use this code anytime there is a reference between HIV stigma and suicide. | Deductive |
|  |  | HIV progression | Use this code if participants describe HIV progression (including but not limited to, death). This also includes opportunistic infections and HIV-related diagnoses (Kaposi sarcoma). Typically, but not always, HIV progression occurs due to stigma and ART nonadherence. | Inductive |
|  |  | Death | Use this code when there is a reference that HIV stigma can cause an unspecified death (i.e. NOT from progression to AIDS or suicide). | Deductive |
|  |  |  |  |  |
|  | Family | Caregiver responsibilities | Use this code to describe presumed caregiver responsibilities of an ALWH. | Deductive |
|  |  | Nontraditional families | Use this code to describe caregiving of ALWH that occurs in a nontraditional family unit (aunts, stepparents, etc). | Deductive |
|  |  |  |  |  |
|  | Disclosure |  | Use this code to describe any general issues around disclosure that do not fit in the below categories. | Inductive |
|  |  | Disclosure family | Use this code to specifically describe disclosure issues within a family unit. | Inductive |
|  |  | Disclosure relationships | Use this code to describe issues of disclosure within the context of romantic relationships and navigating sexual relations. | Inductive |
|  |  | Maintaining secrecy | Use this code when participants talk about the importance of keeping HIV status private/secret. | Deductive |

| \| **Theme** \| **Subtheme** \| **Code** \| **Code definition** \| **Code type** \| \| --- \| --- \| --- \| --- \| --- \| \| **Depression** \| General \| Understanding of depression \| Use this code to describe how participants define depression. \| Deductive \| \|  \|  \| Mphatso responses \| Use this code when participants have rich responses to the Mphatso vignette. \| Deductive \| \|  \|  \| Symptoms of depression \| Use this code to describe symptoms of depression that ALWH experience. \| Deductive \| \|  \|  \| Somatic symptoms (sub-code) \| Use this code to specifically describe somatic symptoms of depression. These are things that you feel in your body. \| Inductive \| \|  \|  \| Causes of depression \| Use this code to describe identified causes of depression. \| Deductive \| \|  \|  \| No DS \| Use this code if participants say they have not experienced depression stigma. \| Inductive \| \|  \|  \| Vulnerability factors \| Use this code when participants describe other social determinants of health (SDOH) that may influence how depression is experienced by ALWH. \| Inductive \| \|  \|  \|  \|  \|  \| \|  \| DS drivers \|  \| Use this code when talking about behaviors that drive stigma around depression. \| Deductive \| \|  \| DS stereotypes \|  \| Use this code for stereotypes and prejudices not otherwise listed below. \| Inductive \| \|  \|  \| Uses substances \| Use this code about assumptions that depressed adolescents are using cannabis or other substances. \| Inductive \| \|  \|  \| Weak \| Use this code for the stereotype that a depressed adolescent is weak. \| Deductive \| \|  \|  \| Selfish \| Use this code for the stereotype that a depressed adolescent is selfish, arrogant, or rude. \| Inductive \| \|  \|  \| Mad or crazy \| Use this code for the stereotype that a depressed adolescent is mad or crazy (with or without a reference to cannabis use). \| Inductive \| \|  \|  \| Dangerous or unpredictable \| Use this code for the stereotype that a depressed adolescent is dangerous or unpredictable. \| Deductive \| \|  \| DS manifestations \|  \| Use this code to describe experienced, witnessed, or anticipated stigma resulting from depression. \| Inductive \| \|  \| Internalized stigma \|  \| Use this code to describe internalized stigma (self-stigma) that results from depression. \| Inductive \| \|  \| Associated stigma \|  \| Use this code to describe stigma experienced by someone else due to association with an adolescent with depression. \| Inductive \| \|  \| Negative consequences \|  \| Use this code to describe negative consequences from depression stigma not otherwise listed below. \| Inductive \| \|  \|  \| Decreased HIV care engagement \| Use this code to describe decreased HIV care engagement resulting from depression stigma. \| Inductive \| \|  \|  \| Isolation \| Use this code to describe pervasive physical/social isolation due to depression stigma. This can be self-isolation or public's isolation but is more consequential than just physical distancing. \| Inductive \| \|  \|  \| Suicidality \| Use this code when describing suicide as a potential negative outcome from depression. \| Inductive \| \|  \| Settings \|  \| Use this code to describe depression stigma experienced in a discrete setting that is not otherwise included. \| Inductive \| \|  \|  \| Home \| Use this code to describe stigma from depression that occurs specifically in home settings and within the family unit. This applies for parents, stepparents, and siblings, or other people residing in the home. \| Inductive \| \|  \|  \| Community \| Use this code for stigma from depression that occurs in community settings, or for broad/unspecified community perceptions of ALWH. Community settings include church, sports, market, and other village settings. \| Inductive \| \|  \|  \| School \| Use this code to describe stigma from depression that occurs in school settings. This can include from teachers and other peers. \| Inductive \| \|  \|  \| Healthcare \| Use this code to describe stigma from depression that occurs in healthcare settings like the clinic or hospital. This can refer to stigma from healthcare workers, other patients, or how the built environment facilitates stigma. \| Inductive \| |
| --- | --- | --- | --- | --- | --- | --- | --- | --- | --- | --- | --- | --- | --- | --- | --- | --- | --- | --- | --- | --- | --- | --- | --- | --- | --- | --- | --- | --- | --- | --- | --- | --- | --- | --- | --- | --- | --- | --- | --- | --- | --- | --- | --- | --- | --- | --- | --- | --- | --- | --- | --- | --- | --- | --- | --- | --- | --- | --- | --- | --- | --- | --- | --- | --- | --- | --- | --- | --- | --- | --- | --- | --- | --- | --- | --- | --- | --- | --- | --- | --- | --- | --- | --- | --- | --- | --- | --- | --- | --- | --- | --- | --- | --- | --- | --- | --- | --- | --- | --- | --- | --- | --- | --- | --- | --- | --- | --- | --- | --- | --- | --- | --- | --- | --- | --- | --- | --- | --- | --- | --- | --- | --- | --- | --- | --- | --- | --- | --- | --- | --- | --- | --- | --- | --- | --- | --- | --- | --- | --- | --- |
| \| **Theme** \| **Subtheme** \| **Code** \| **Code definition** \| **Code type** \| \| --- \| --- \| --- \| --- \| --- \| \| **Intersectional Stigma (IS)** \| Differences \|  \| Use this code to describe differences in experiences between HIV and depression stigma. \| Deductive \| \|  \| Similarities \|  \| Use this code to describe similarities in experiences between HIV and depression stigma. \| Deductive \| \|  \| IS experiences \|  \| Use this code to describe the experienced, anticipated, or witnessed intersectional stigma of living with both HIV and depression. \| Deductive \| \|  \| No IS \|  \| Use this code if participants say they have not experienced intersectional stigma. \| Inductive \| \|  \|  \|  \|  \|  \| \|  \|  \|  \|  \|  \| \| **Coping** \| HIVS coping \|  \| Use this code when talking about coping with HIV stigma ONLY. Use this code if it does not fit into one of the categories below \| Inductive \| \|  \|  \| Social support \| Use this code to describe social support as a coping mechanism (from peers, family, etc). \| Deductive \| \|  \|  \| Religion \| Use this code to describe references to faith/religion/church as a coping mechanism. \| Deductive \| \|  \|  \| Acceptance of diagnosis \| Use this code when participants talk about acceptance of HIV diagnosis as an important coping mechanism. \| Deductive \| \|  \|  \| Distraction \| Use this code when participants talk about distraction as a coping mechanism. \| Deductive \| \|  \|  \| Negative coping mechanisms \| Use this code when talking about negative or maladaptive coping mechanisms. \| Deductive \| \|  \|  \| How to help \| Use this code to describe ideas of how we can help adolescents with HIV better cope with stigma. \| Deductive \| \|  \|  \|  \|  \|  \| \|  \| DS coping \|  \| Use this code when talking about coping with depression (and associated stigma) ONLY. Use this code if it does not fit into one of the categories below. \| Inductive \| \|  \|  \| Social support \| Use this code to describe social support as a coping mechanism (from peers, family, etc). \| Deductive \| \|  \|  \| Religion \| Use this code to describe references to faith/religion/church as a coping mechanism. \| Deductive \| \|  \|  \| Acceptance of diagnosis \| Use this code when participants talk about acceptance of HIV or depression diagnosis as an important coping mechanism. \| Deductive \| \|  \|  \| Distraction \| Use this code when participants talk about distraction as a coping mechanism. \| Deductive \| \|  \|  \| Negative coping mechanisms \| Use this code when talking about negative or maladaptive coping mechanisms. \| Deductive \| \|  \|  \| How to help \| Use this code to describe ideas of how we can help adolescents with depressions. \| Deductive \| \|  \|  \|  \|  \|  \| \|  \| IS coping \|  \| Use this code when talking about coping with intersectional stigma ONLY (living with both HIV and depression). This is the last section of the guide. Use this code if it does not fit into one of the categories below. \| Inductive \| \|  \|  \| Social support \| Use this code to describe social support as a coping mechanism (from peers, family, etc). \| Deductive \| \|  \|  \| Religion \| Use this code to describe references to faith/religion/church as a coping mechanism. \| Deductive \| \|  \|  \| Acceptance of diagnosis \| Use this code when participants talk about acceptance of HIV or depression diagnosis as an important coping mechanism. \| Deductive \| \|  \|  \| Distraction \| Use this code when participants talk about distraction as a coping mechanism. \| Deductive \| \|  \|  \| Negative coping mechanisms \| Use this code when talking about negative or maladaptive coping mechanisms. \| Deductive \| \|  \|  \| Resiliency factors \| Use this code when someone describes what makes one person more resilient than another. \| Deductive \| \|  \|  \| How to help on individual level \| Use this code to describe ideas of how we can help ALWH address IS stigma on the individual level (i.e. addressing self-stigma). \| Inductive \| \|  \|  \| How to help on public level \| Use this code to describe ideas of how we can address IS stigma on the public level. \| Inductive \| |
|  |
|  |
|  |
|  |
|  |
|  |
|  |
|  |
|  |
|  |
|  |
